# ISDB: Interaction Sentence Database

**DOI:** 10.1186/1756-0500-3-122

**Published:** 2010-05-03

**Authors:** Michael A Bauer, Robert E Belford, Jing Ding, Daniel Berleant

**Affiliations:** 1 Department of Information Science, University of Arkansas at Little Rock, 2801 S. University Ave, Little Rock, AR, 72204, USA; 2 University of Arkansas for Medical Sciences, 4301 W. Markham St., Little Rock, AR, 72205, USA; 3 Department of Chemistry, University of Arkansas at Little Rock, 2801 S. University Ave, Little Rock, AR, 72204, USA; 4 Ohio State University Medical Center, 410 W. 10th Avenue, Columbus, OH, 43210, USA

## Abstract

**Background:**

Rapid growth in the scientific literature available on-line continues to motivate shifting data analysis from humans to computers. For example, greater knowledge of sentence characteristics indicative of interaction between two biological entities is needed to aid in the creation of better-performing information extraction tools for effectively using this rich body of information.

**Findings:**

The Interaction Sentence Database (ISDB) allows users to retrieve sets of sentences fitting specified characteristics. To support this, a database of sentences from abstracts in MEDLINE was created. The sentences in the database all contain at least two biomolecule terms and one interaction-indicating term. A web interface to the database allows the user to query for sentences containing an interaction-indicating term, a single biomolecule name, or two biomolecule names, as well as for a list of biomolecules co-occurring with a given biomolecule in at least one sentence.

**Conclusions:**

The system supports researchers needing conveniently available sets of sample sentences for corpus-based research on sentence properties. It also illustrates a model architecture for a sentence-based retrieval system which would be useful to people seeking information and knowledge on-line. ISDB can be freely accessed over the Web at http://bioinformatics.ualr.edu/cgi-bin/services/ISDB/isdb.cgi, and the processed database will be provided upon request.

## Introduction

Traditionally, information obtained for biological research is stored as text in journals. When a paper is submitted to a journal it is often left to curators to pull information from the text into specialized resources for researchers [1]. Such specialized resources include interaction, biomolecule complex and pathway databases. As an alternative to reading a stack of papers, a simple query of a specialized database can then potentially return the information of interest. The development of better automated literature mining tools is essential if we are to take full advantage of the daunting amount of available information.

Information extraction is the process of pulling facts from text [2]. Accurate software tools for information extraction are needed to automate the process of pulling these facts from text to populate specialized databases. The fragmentation of scientific expertise and the resulting highly specialized fields leads to disconnects among researchers [3,4]. Possible connections go undiscovered due to lack of researchers with enough cross-disciplinary knowledge to make those connections [5]. Automated information extraction tools promise to address this problem by gathering facts from different disciplines and feeding other tools that find connections which might otherwise be overlooked.

The motivation for this project was to create a tool that would give text mining researchers flexible access to a corpus of sentences relevant to studying how biomolecular interactions are described. The Interaction Sentence Database (ISDB) is the result. It is processed from the MedRep http://bioinformatics.ualr.edu/dan/medrep/ repository and contains sentences containing at least two biomolecule names and one of about 440 interaction-indicating terms.

The term co-occurrence based approach was chosen because of its high recall and computational efficiency. Because the existence of co-occurring biomolecules in the sentence is the criterion for its retrieval, the threshold for retrieval of sentences describing their interaction is low, hence recall is high.

Although relevant sentences that refer *anaphorically *to one of the biomolecules will not be retrieved, Ding et al. (2002)[6] found that retrieving them would boost recall by only a modest 8%. The same study found a precision of 0.638 for retrieved sentences. Precision is intrinsically limited for co-occurrence based approaches because a sentence that is retrieved due to containing biomolecule terms A and B might not describe them as interacting (even if they do interact). Another sentence might contain terms C and D which do not interact but are both present in the same sentence for some other reason, such as both interacting with some other biomolecule. This exemplifies the challenge of text mining for biomolecular interactions.

The sentences were obtained from MEDLINE abstracts. A dictionary of approximately 40,000 unique biomolecules obtained from the LIGAND, ENZYME, and Swiss-Prot databases was developed and used to pull these sentences from MEDLINE to construct the MedRep repository. A sentence was defined as a title, or a string between two neighboring sentence boundaries. A boundary was defined as the start of an abstract, the end of an abstract, or one or more spaces preceded by a period and followed by a capital letter. Ultimately 4,404,697 such sentences were cataloged that each contain at least two biomolecules and one interaction-indicating term. 443 different interaction indicating terms are recognized, like *activate*, *inhibits*, etc., and counting different grammatical forms of a term separately. Each sentence is stored with the PubMed ID of the abstract it is from.

ISDB users do not have to sift through MEDLINE or other on-line resources to find sentences for their research but instead can use ISDB to efficiently obtain them. Researchers can retrieve data sets of sentences that contain a certain interaction term, biomolecule name, or a specific pair of biomolecule names. With those data sets they can, for example, investigate statistical and natural language processing techniques on sentences to better extract information from them about biomolecular interactions. The tool can also return a set of biomolecules instead of sentences. For example, if name 'A' is input by the user, a list of other biomolecules co-occurring with 'A' in at least one other sentence can be returned. Finally the tool demonstrates a model architecture for specialized, sentence-finding search engines for biological texts.

The ISDB *architecture *also supports a more general audience of users, those who seek information about biomolecular interactions, rather than just text mining researchers. However, because ISDB is not continually updated as new papers are added to MEDLINE, the ISDB system itself is thus mostly oriented toward those who need sample sentences to analyze. Its architecture can however serve as a model for constructing a system that does update dynamically as new papers are published but is otherwise similar to ISDB. Such a system would be valuable to the more general audience.

## Related work

Using sentences as annotations in a publicly available resource is an established approach. MedMiner [7] was one of the earlier such systems and represented a significant advance, with several functionalities including sentence-based retrieval not focused specifically on interactions. MedMiner was oriented toward information users rather than text mining researchers, and did not support mining researchers directly as ISDB does. A well-known example in current use is GeneRIF (**Gene R**eference **I**n **F**unction), a curated resource of short texts of 255 characters or less http://www.ncbi.nlm.nih.gov/projects/GeneRIF/GeneRIFhelp.html, provided by NCBI. However GeneRIF specifically addresses gene function, not biomolecular interaction, and is not provided with a text mining researcher-friendly interface although it itself is the subject of a current of research. PathBinderH [8] provides access to sentences of a wide variety. It demonstrates retrieval based on inheritance within the Linnaean taxonomy, rather than interactions.

There are other relevant systems that aid researchers in traversing the PubMed literature and visualizing connections in the literature. Two such system are iHOP [9] and Chilibot [10]. The iHOP system allows the user to navigate through the literature using proteins and genes as hyperlinks among sentences and abstracts. Chilibot is more interaction focused. It builds graphical representations of connections between user provided entities. The relationships in the network are linked back to supporting material, usually sentences, that contain both terms. These tools are designed for biological researchers, whereas the design of ISDB was motivated by the need for a tool for text mining researchers who need sets of sentences to analyze (e.g. [6,11]).

PreBIND [12] was an ambitious project that included the Textomy system, supported BIND, and is now available as BINDplus, a commercial product. While sentences were originally made available as annotations to interactions, the focus is on the actual interactions themselves rather than the sentences, and on supporting biological researchers rather than text mining research, in contrast to ISDB. There are other corpora and annotated sentence data sets that describe interactions, such as GENIA [13] and BioInfer[14]. What distinguishes the ISDB system from these is the support it provides for research on text mining of interactions. ISDB's database is constructed to allow three categories of searches for sentences containing particular biomolecules or interaction-indicating terms. It supports different types of queries related to biomolecules and interaction-indicating terms, and provides a convenient web interface.

## Graphical User Interface

**Figure 1:**
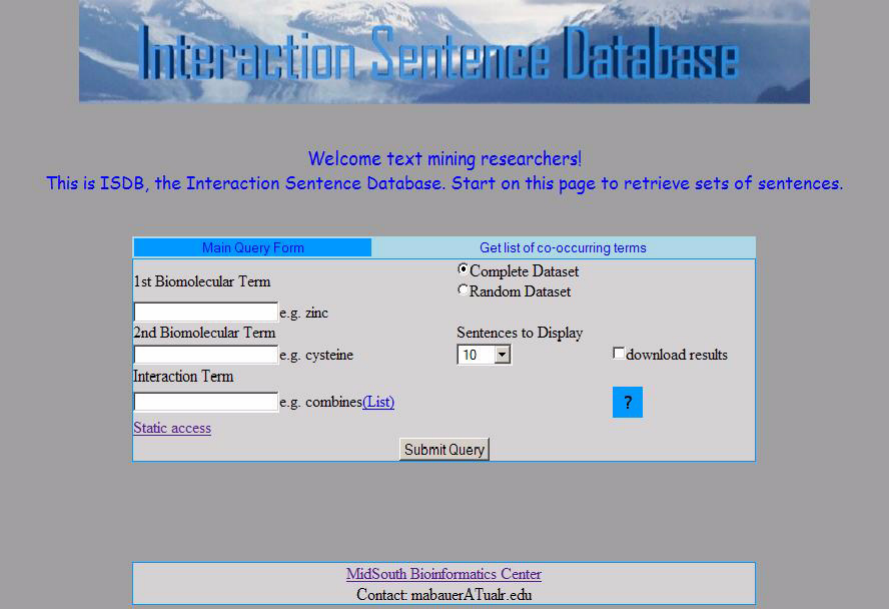
**Main query form**. Three types queries can be performed using this form. The appropriate query is performed based on which text boxes are filled upon submission of the form. The first is a simple query for retrieving sentences that contain either a certain interaction-indicating term or a biomolecule, depending on which text box is used. The second type of query is for sentences that contain two biomolecule name terms. There are two text input fields for the two biomolecule name terms of interest. The third type of query occurs when all text boxes are filled. This query takes two biomolecules and one interaction-indicating term, and returns sentences that contain the three terms. There is also an option to return a random set of *N *sentences.

A web interface allows the user to query for different sets of sentences that fit particular criteria http://bioinformatics.ualr.edu/cgi-bin/services/ISDB/isdb.cgi. There are four different types of queries, three of which are input from the same screen. The appropriate query is performed based on which text boxes are filled upon submission of the form (Figure 1).

• The first is a simple query for retrieving sentences that contain either a certain interaction-indicating term or a biomolecule, depending on which text box is used. There is also an option to return a random subset of N of these sentences to use as a test set, where the user sets the value of N.

• The second type of query is for sentences that contain two biomolecule name terms. There are two text input fields for the two biomolecule name terms of interest. This type of query also has the option to return a random subset of N sentences matching the specified query.

• The third type of query occurs when all text boxes are filled. This query takes two biomolecules and one interaction-indicating term, and returns sentences that contain the three terms. Again the option to return a random subset of N sentences matching the specified query is available.

• The fourth query type finds biomolecule names that co-occur in one or more sentences with the query biomolecule name. A list of other biomolecule names co-occurring with the queried one is returned.

**Figure 2:**
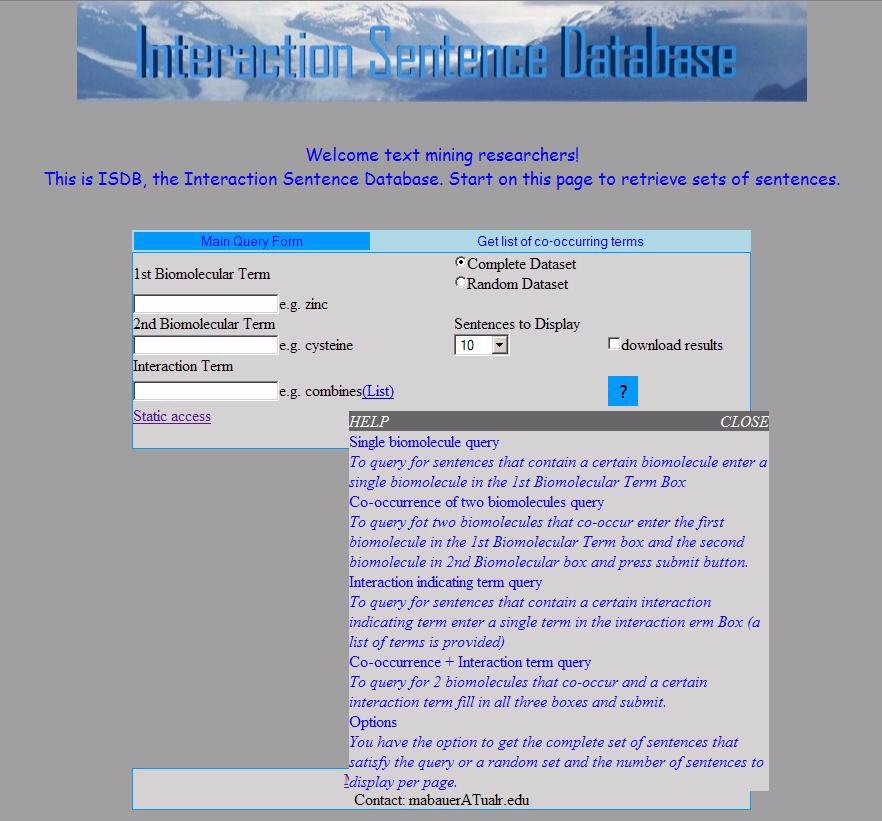
**Tool tip to help explain the query form**. Tool tips are provided to explain what to input in the text boxes and to explain the different query possibilities. This tool tip is accessed by mousing over the question mark in the lower right corner of the form.

**Figure 3:**
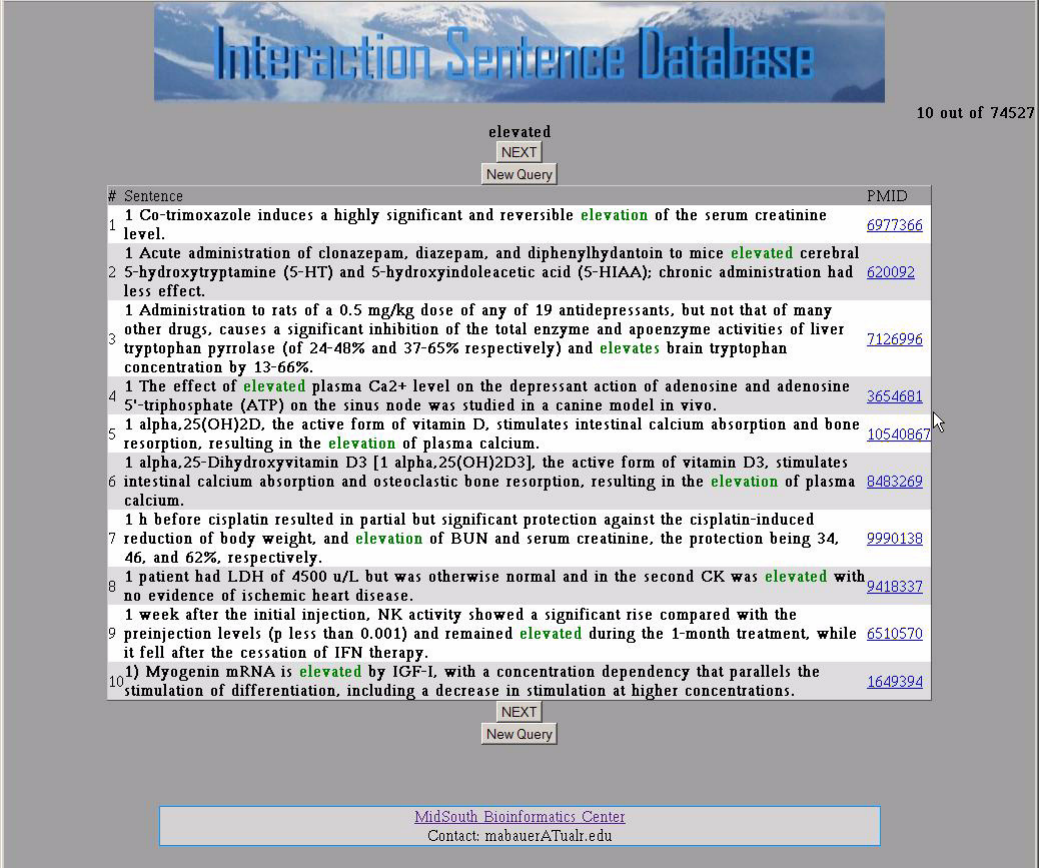
**Returned sentences**. An example of a list of sentences returned by a query for the interaction-indicating term 'elevated.' The returned results show 10 sentences out of 74,527 that contain the term. The terms are marked in green. Note that 'elevation' is also included, showing that different forms of the base term are automatically included in the query. The hyperlink to the left of each sentence is to the abstract in PubMed that contains it.

**Figure 4:**
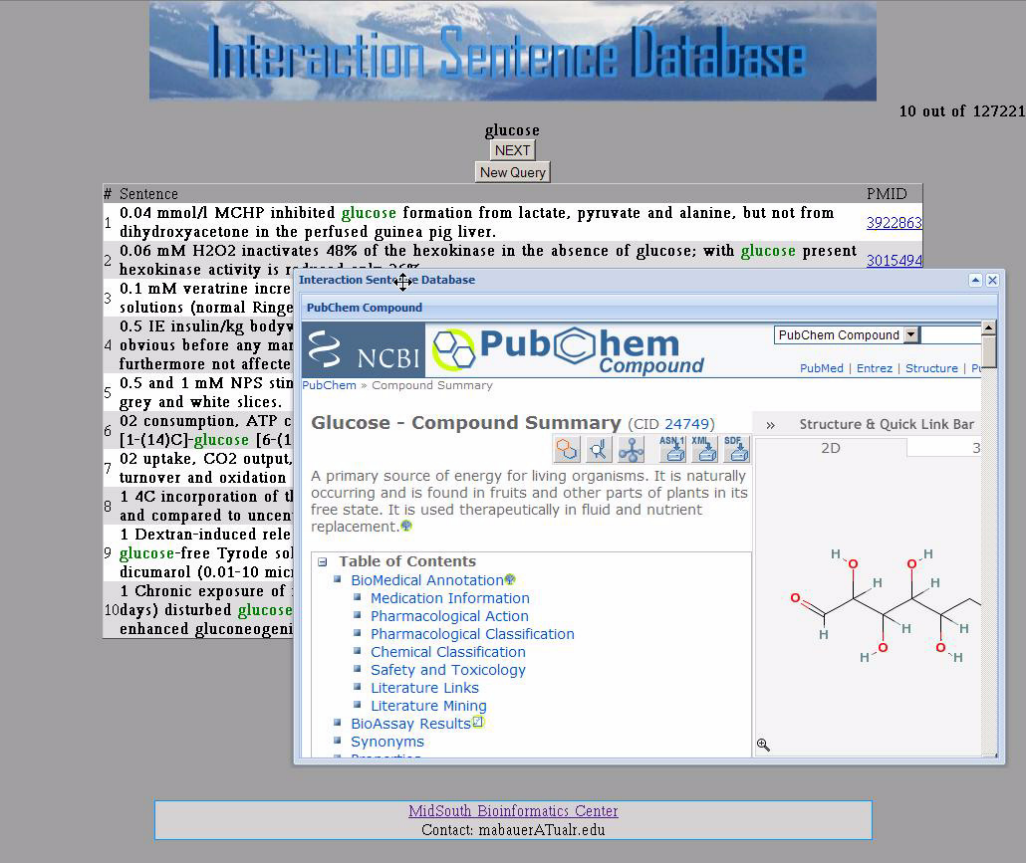
**Screen shot of the PubChem Compound search result window**. A simple search of PubChem is performed when a marked biomolecule term is selected and the results are shown in a pop up window.

For queries that include an interaction-indicating term, that term is automatically expanded to include other inflections of that term. For example, the query term 'activate' is expanded to include 'activated,' 'activates,' 'activating,' 'activation,' and 'activator.' When no sentences are retrieved, because one or more of the terms was not recognized, the system lets the user know the number of sentences that can be found based on the terms that are recognized. Tool tips are provided to explain how to fill out the query form (Figure 2). The input screen for each type of query can be reached from the navigation bar at the top of each form. The user can also choose the number of sentences to view per page. Example output can be seen in Figure 3. The terms in the query are colored in the results display. A left click on a biomolecule term initiates a search of NCBI's PubChem Compound database with the results appearing in a window (Figure 4).

## Conclusion

This database gives researchers access to a large data set of sentences that have at least two biomolecule names and one interaction-indicating term. This database provides a graphical user interface that allows performing four different types of queries. The retrieved sentences can be used as data sets for research on text mining of biomedical texts. The system also demonstrates a model architecture for sentence-based biological interaction search engines.

## Availability and Requirements

The ISDB system conforms to the following list.

• **Project name**: ISDB (Interaction Sentence Database)

• **Web interface**: http://bioinformatics.ualr.edu/cgi-bin/services/ISDB/isdb.cgi

• **Project home page**: http://bioinformatics.ualr.edu/ISDB/

• **Computer system requirements**: ISDB runs on common Web browsers

• **License**: freely available

## Competing interests

The authors declare that they have no competing interests.

## Authors' contributions

MB designed and developed the graphical user interface and the database. The text of the paper was written by MB and DB. DJ created the original MedRep repository which was used as a starting point for the ISDB project. RB directs the WikiHyperGlossary system integration and compatibility effort. All authors have read and approved the final manuscript.
